# Root imaging showing comparisons in root distribution and ontogeny in novel *Festulolium* populations and closely related perennial ryegrass varieties

**DOI:** 10.1002/fes3.145

**Published:** 2018-10-02

**Authors:** Mike W. Humphreys, John H. Doonan, Roger Boyle, Anyela C. Rodriguez, Christina L. Marley, Kevin Williams, Markku S. Farrell, Jason Brook, Dagmara Gasior, Dimitra Loka, Rosemary P. Collins, Athole H. Marshall, Debbie K. Allen, Rattan S. Yadav, Jennifer A. J. Dungait, Phil Murray, John A. Harper

**Affiliations:** ^1^ IBERS Aberystwyth University, Gogerddan Aberystwyth Ceredigion UK; ^2^ Genetics and Breeding The John Bingham Laboratory National Institute of Agricultural Botany (NIAB) Cambridge UK; ^3^ Sustainable Agriculture Sciences, Rothamsted Research Okehampton Devon UK

**Keywords:** ecosystem services, *Festulolium*, *Lolium perenne*, root ontogeny, root phenomics

## Abstract

The incorporation of new sophisticated phenotyping technologies within a crop improvement program allows for a plant breeding strategy that can include selections for major root traits previously inaccessible due to the challenges in their phenotype assessment. High‐throughput precision phenotyping technology is employed to evaluate root ontogeny and progressive changes to root architecture of both novel amphiploid and introgression lines of *Festulolium* over four consecutive months of the growing season and these compared under the same time frame to that of closely related perennial ryegrass (*L. perenne*) varieties. Root imaging using conventional photography and assembled multiple merged images was used to compare frequencies in root number, their distribution within 0–20 and 20–40 cm depths within soil columns, and progressive changes over time. The *Festulolium* hybrids had more extensive root systems in comparison with *L. perenne*, and this was especially evident at depth. It was shown that the acquisition of extensive root systems in *Festulolium* hybrids was not dependent on the presence of an entire *Festuca* genome. On the contrary, the most pronounced effect on root development within the four *Festulolium* populations studied was observed in the introgression line Bx509, where a single small genome sequence from *F. arundinacea* had been previously transferred onto its homoeologous site on the long arm of chromosome 3 of an otherwise complete *L. perenne* genome. This demonstrates that a targeted introgression‐breeding approach may be sufficient to confer a significant improvement in the root morphology in *Lolium* without a significant compromise to its genome integrity. The forage production of Bx509 was either higher (months 1–3) or equivalent to (month 4) that of its *L. perenne* parent control demonstrating that the enhanced root development achieved by the introgression line was without compromise to its agronomic performance.

## 1 INTRODUCTION

Grass varieties developed and marketed for agricultural use within Europe have hitherto been selected solely on their forage quality, yield, and persistence with little or no regard given to their root growth or architecture. The selection strategies used for plant breeding in the UK, usually under high nutrient supply over the last 100 years, have undoubtedly led to significant crop advancement in terms of forage yield (Humphreys et al., 2006). However, plant breeding strategies should become more holistic taking account not only of the potential for grass crop production but also the impact these grasses make on their surrounding environment, and in particular, the role of plant–soil interactions in ecosystem service provision (Marshall, Collins, Humphreys, & Scullion, 2015).

Perennial ryegrass (*Lolium perenne* L.) and Italian ryegrass (*Lolium multiflorum* L.), grown in temperate locations and subject to relatively moderate summer and winter stresses, provide excellent growth and forage quality traits suitable for use in livestock agriculture. Using conventional breeding technologies, closely related fescue (*Festuca*) species, while inferior to *Lolium* in terms of their agronomic performance, have greater resilience when subjected to winter and summer stresses. Therefore, through intergeneric hybridization, they provide opportunities to combine in a single genotype the complementary traits found in species of both grass genera. As a consequence, *Festulolium* (*Lolium* spp. × *Festuca* spp. hybrids) varieties with favorable agronomic and persistency traits are marketed increasingly as a response to increasing episodes of more extreme weather events, frequently attributed to climate change (Ghesquière, Humphreys, & Zwierzykowski, 2010; Humphreys, O'Donovan, Farrell, Gay, & Kingston‐Smith, 2014). Also, there are increasing requirements to ensure cost‐effective forage production is achieved with a lower environmental footprint.

Improved phenotyping technologies now allow direct observation and measurement of root ontogeny and architecture, as well as the interactions of roots with soil, and are now increasingly incorporated as selection criteria within plant breeding programs. Root system architecture describes the spatial arrangement of roots within the soil and plays a significant role in crop performance (Marshall et al., 2015). As well as providing plant anchorage, roots are essential in the uptake of nutrients, especially nitrogen (N) and phosphorus (P), and water from the soil. As with all plants, grasses have evolved different capabilities to capture these resources, and their abilities for uptake and subsequent use are major factors in achieving optimal forage yield. Relevant root architectural traits include rooting depth, density, and diameter. Physiological root traits of relevance are respiration and nutrient uptake as well as the extent and nature of release of root exudates combined with subsequent interactions with soil biota, especially mycorrhizae (Bardgett, Mommer, & De Vries, 2014). Many reports have shown root architecture as being plastic and shaped by interactions between genotype and components of the local soil environment, which include nutrient and water localization, the soil microbiome, and the physical properties of soil (Rogers et al., 2016).

There is a growing appreciation of the multifunctional properties of grasslands and the need to ensure their attributes both as providers of healthy livestock feed and ecosystem service is incorporated as targets within the breeding programs of forage grasses and legumes. Grasslands have a fundamental role in ecosystem provision due to their significant land cover, biodiversity, perpetuity over many consecutive years, and location in upland and marginal areas. Through their root–soil interactions, grasslands play a major role in river catchments, regulating both rainwater capture and its subsequent release (Humphreys et al., 2014; MacLeod et al., 2013).

The development of new high‐throughput genomic and phenotyping technologies and their incorporation into multidisciplinary research enables a new holistic approach to variety development. An example is the BBSRC‐LINK‐funded Program “SureRoot” (http://www.sureroot.uk/) where, in addition to assessing agronomic performance, modified grass and clover root designs either used alone or as mixtures are seen to aide resilience to the onset of drought. They also provide porous soil structures that assist soil water retention following onsets of heavy rainfall to mitigate runoff and flood conditions. Relevant to the new plant breeding approach has been the emergence of novel *Festulolium* hybrids that combine the good agronomic attributes of *Lolium* species with the deep rooting and stress resistance found in various close relatives of the broad‐leaved fescue (*Festuca*) subgenus *Schedonorus* (Humphreys et al., 2014; Kopecky et al., 2016) and as hybrids provide novel opportunities for additional ecosystem service (Humphreys et al., 2014; MacLeod et al., 2013).

New plant breeding targets that necessitate detailed measures of underground root growth and their changes throughout a growing season require the incorporation of novel methodologies and technologies in plant phenotyping. Reported here is a method for the reliable and objective measurement of root growth, incorporating image capture and extraction protocols sufficient to capture and quantify dynamic changes in root development, distribution, and turnover in forage grasses over a sequential 4‐month period of the growing season.

As proof of principle, populations of diploid and tetraploid *L. perenne* varieties were grown under the same growth conditions and time frame as representative populations of two diploid *Festulolium* introgression lines selected for their drought resistance and two amphiploid *Festulolium* hybrid populations.

## 2 MATERIALS AND METHODS

### 2.1 Plant material

Commercial varieties within the *Lolium/Festuca* genome complex are maintained as outbreeding populations and so representative samples from each variety are required to ascertain the extent of intra‐ and interspecific differences in morphological traits such as root architecture and to determine the mean trait values for each variety or advanced breeding population. The six populations used were as follows: 1) *L. perenne* cv AberStar (2x), 2) *L. perenne* cv AberBite (4x), 3) *Festulolium* drought‐tolerant introgression line Bx509 (2x), 4) *Festulolium* drought‐tolerant introgression line Bx510 (2x), 5) *Festulolium* amphiploid Bx511 (*L. perenne x F. mairei*) (4x), and 6) *Festulolium* amphiploid Bx514 (*L. perenne* x *F. arundinacea var glaucescens*) (4x).

The drought‐tolerant introgression lines, Bx509 and Bx510, were backcross derivatives where fescue genes from *F. arundinacea* (in Bx509) and from *F*. *arundinacea var glaucescens* (in Bx510) had been transferred onto two alternative locations on chromosome 3 in an otherwise complete genome of *L. perenne* cv AberStar (2x) (Humphreys et al., 2017; Marshall et al., 2015). The derivations of the two *Festulolium* amphiploid populations Bx511 and Bx514 and their potential agronomic value in livestock agriculture are described in Humphreys et al. (2014). In both cases, fescue genomes were combined with those derived from National Listed tetraploid *L. perenne* varieties. For comparisons of root ontogeny and architecture in diploid Bx509 and Bx510, *L. perenne* cv AberStar (2x) was included as it was used in their development. For comparisons of root ontogeny and architecture in allotetraploid Bx511 and Bx514, *L. perenne* cv AberBite (4x), a current high‐performing National Listed tetraploid *L. perenne* variety used as a control in *Festulolium* field trials (Humphreys et al., 2014) and sharing similar *Lolium* genetic background as Bx511 and Bx514, was considered appropriate.

### 2.2 Experimental design

For each plant genotype in each population, three replicate clonal sets, each comprising five single tillers of equal maturity, size, and vigor, were selected. The six grass populations used in the root growth analysis (Bx509, Bx510, Bx511, Bx514, AberStar, and AberBite) each comprised 20 randomly selected individual plant genotypes. The three clonal sets of each genotype were planted in transparent perspex soil columns (12 × 12 × 50 cm) filled with Levingtons F2 potting compost (i.e., 3 × 20 soil columns/grass population). The five clonal tillers for each soil column were arranged with four occupying separate corners and the remaining tiller located in a central position. For replicate sets of each grass population, soil columns were arranged randomly and subsequently close‐packed with the sides of the outer soil columns of each block wrapped with black horticultural fabric to exclude light from the roots.

The grass tillers were installed in well‐watered compost in the soil columns on 3 March 2015 and allowed to establish initially under lights in a glasshouse with supplementary heating and lighting (set to 20°C; 16 hr light). On the day preceding root scanning, root columns were transferred from the glasshouse in which they were maintained to the National Plant Phenomics Centre (NPPC) at IBERS for scanning to monitor root development over four consecutive months using LemnaTec technologies (as illustrated in Marshall et al., 2015). Root scans were undertaken on 14 April 2015, 12 May 2015, 09 June 2015, and 16 July 2015. Just prior to each root scanning, all forage above 4 cm from the soil surface was removed from each root column and subsequently dried (80°C, 24 hr). The mean dry matter yield (DMY) per cut/grass genotype (g) was determined. From these weights, the overall mean DMY for the six grass populations/cut was determined based on the means of three clonal replicates of 20 plant genotypes/grass population.

Following each monthly root scan, soil columns containing the six grass populations were returned to the glasshouse and reassembled as blocks within their replicate groups, all columns randomly arranged in their respective block, with the sides of the outer soil columns wrapped with black horticultural fabric (as described above). All soil columns were maintained in close proximity and the grasses allowed to continue growth under ambient conditions without recourse to any further supplementary lighting and heating throughout the 4 months of their assessment. All plants in the soil columns were watered regularly as required to maintain optimal growth. No supplementary fertilizer was provided throughout the experiment.

### 2.3 Data acquisition and analysis

Aboveground growth was removed to 4–5 cm above soil level (in accordance with standard IBERS procedures for simulated grazing, e.g., Humphreys, Harper, Armstead, & Humphreys, 2005) prior to each root scan. Three high‐resolution images of soil columns using a high‐vis camera system (http://www.lemnatec.com) were captured at alternative heights and subsequently aligned and combined to provide data on visible root frequency/column side. Subsequently, each soil column was rotated 90° to allow further root imaging in turn of all four sides. The twelve root images from each soil column were merged to provide a total visible root score for every plant genotype (Marshall et al., 2015).

Mean root scores for every plant genotype within each of the six plant populations for four consecutive monthly time‐points were determined based in every instance on data obtained from three clonal replicate sets in their respective soil columns. Using data from individual genotypes, overall comparisons between the six plant populations at each monthly time‐point were achieved through calculations of overall population means based on 20 randomly selected genotypes considered representative of each population.

For all plant populations, mean root frequencies were compared at two soil depths. Mean root frequencies were determined based on their detection within a 40 cm region within each 50 cm soil column represented the soil area immediately beneath the aboveground plant tiller bases to the upper surface of the carriages used for transporting the soil columns along the conveyor into the NPPC imaging chamber. Root frequencies were compared at 0–20 and 20–40 cm depths. Data were normalized, and spurious values set to missing and then imputed using the means for root distribution within each plant population. Root ontogeny was analyzed using two‐way ANOVA on the main effects: genotype, mean root numbers recorded at 0–20 and 20–40 cm depths, and month of measurement. Tukey's multiple comparison test was used to perform pairwise comparisons for genotype x date and distribution and to confirm significant differences (*p* < 0.05). *p*‐values were adjusted using Bonferroni correction (Bland & Altman, 1995).

Using all available plant materials in the root columns, the mean DMY for each grass genotype for all six grass populations taken at all four cuts immediately prior to the four monthly root scans was compared statistically using standard procedures with the menu‐driven options within GenStat 13.2 for Windows (VSN International Ltd.) software and any significant yield differences between the *Festulolium* populations at each cut compared to their relevant diploid and tetraploid *L. perenne* controls (*p* < 0.05) determined.

## 3 RESULTS

**Figure 1:**
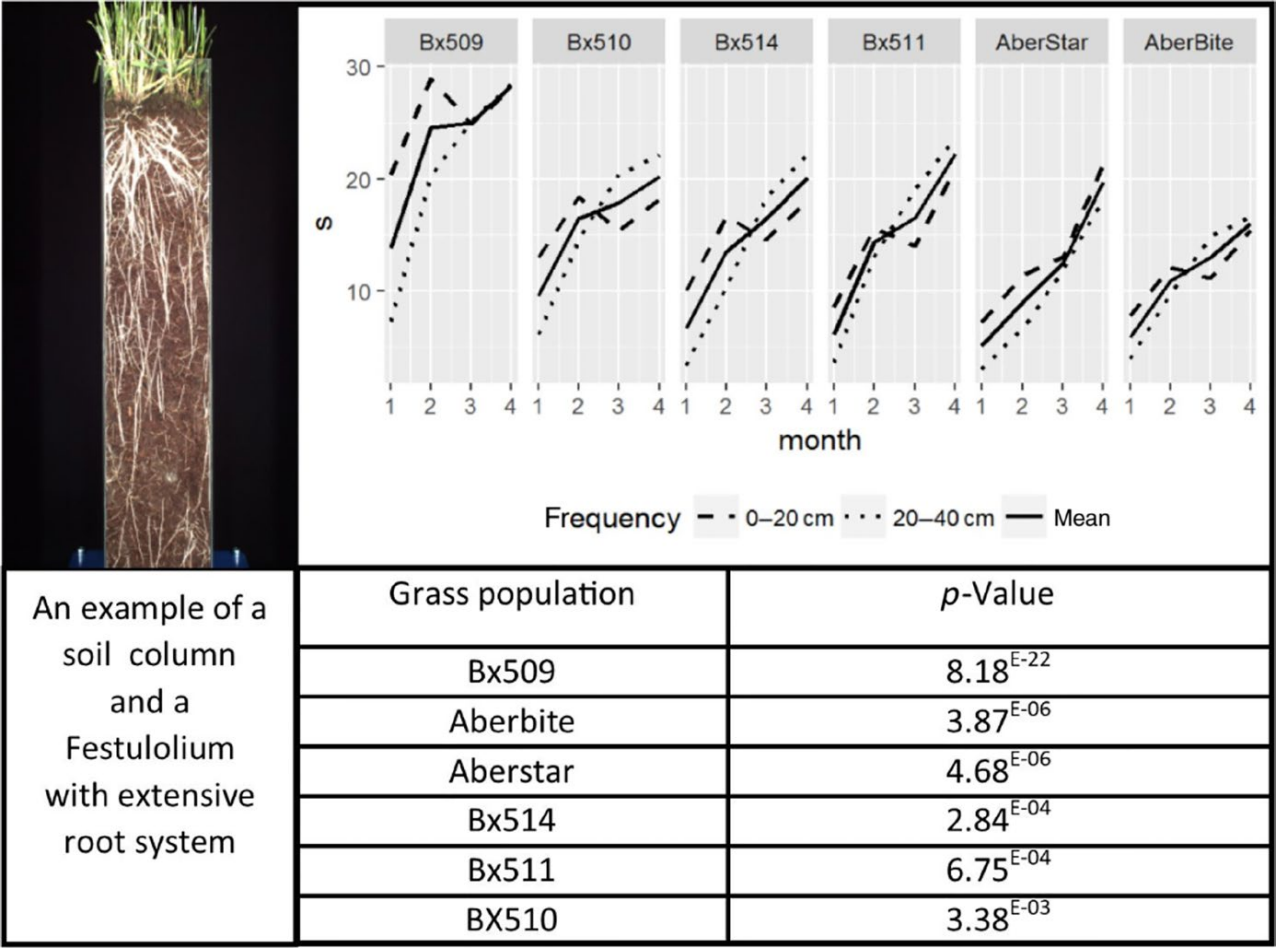
Changes in mean root frequencies (s) over four consecutive months at 0–20 cm (—) and 20–40 cm (….) depths in soil columns filled with potting compost in six glass populations, *L. perenne* cvs AberStar (2×) and AberBite (4×); two drought‐tolerant *Festulolium* introgression lines Bx509 and Bx510 (both 2×), and two *Festulolium* amphiploid hybrids Bx511 (*L. perenne *× *F. mairei*) and Bx514 (*L*. *perenne *× *F. arundinacea var glaucescens*) (both 4×). Overall root mean/population is shown as a solid line. Root frequency/population is based on calculations of mean values taken from merged images acquired from LemnaTec facilities based at the NPPC, IBERS, accumulated from 20 randomly selected plant genotypes/population with each genotype of each population represented as three replicate sets of five clonal tillers/soil column. Total mean root frequency differences, 0–40 cm/month between grass populations calculated using ANOVA (*p* < 0.05). *p*‐values represent overall (depths and time) differences between different grass populations

Significant differences (*p* < 0.05) in root ontogeny between the four *Festulolium* populations and their ryegrass controls were evident when root frequencies were accumulated within the upper (0–20 cm) and lower (20–40 cm) soil profiles (Figure 1; Table 1). Figure 1 illustrates overall population differences in root number and separates root frequency differences taken at 0–20 and 20–40 cm depths within soil columns recorded over four consecutive months. Also provided are population statistical differences between lines based on mean root frequency/month. The *Festulolium* populations increased their root number between months 1 and 2, although for population Bx510 this applied only to the 20–40 cm soil profile. The other three *Festulolium* populations showed increased root frequency at all depths throughout four consecutive months (Table 1). This contrasted with the two ryegrass varieties where no differences in root frequency were evident between months 1 and 3 within the upper soil region, although evidence for enhanced root growth at depth was apparent within the two ryegrass varieties, particularly in AberBite over months 2 and 3. Only *Festulolium* Bx511 and ryegrass variety AberStar had further root increase in month 4 (Table 1). Bx510 was unique among the six grasses in showing no significant (*p* > 0.05) increases in root number within the upper 20 cm between month 1 and month 4.

**Table 1:** Changes in root number (a) in 0–20 cm, (b) in 25–40 cm soil profiles over four consecutive months in *Festulolium* introgression lines Bx509 and Bx 510 (both diploid), *Festulolium* amphiploids Bx511 and Bx514 (both tetraploid), and *L. perenne* cvs AberStar (diploid) and AberBite (tetraploid). Grasses with significantly more roots are indicated (*p* < 0.05*; *p* < 0.01**; *p* < 0.001***, NS = not significant)

Grass	Soil depth (cm)	Month 1 (M1) vs. month 2 (M2)	Month 2 (M2) vs. month 3 (M3)	Month 3 (M3) vs. month 4 (M4)	Month 1 (M1) vs. month 4 (M4)
Bx509	0–20	***	P = 0‐497 (NS)	*p* = 0.929 (NS)	*
	20–40	***	*	*p* = 0.304 (NS)	***
Bx510	0–20	*p* = 0.117 (NS)	*p* = 0.788 (NS)	*p* = 0.836 (NS)	*p* = 0.145 (NS)
	20–40	***	*	*p* = 0.965 (NS)	***
AberStar	0–20	*p* = 0.405 (NS)	*p* = 0.993 (NS)	***	***
	20–40	*p* = 0.421 (NS)	*	**	***
Bx511	0–20	***	*p* = 0.974 (NS)	***	***
	20–40	***	**	*p* = 0.102 (NS)	***
Bx514	0–20	**	*p* = 0.931 (NS)	*p* = 0.457 (NS)	***
	20–40	***	***	*p* = 0.258 (NS)	***
AberBite	0–20	*p* = 0.195 (NS)	*p* = 1 (NS)	*p* = 0.202 (NS)	***
	20–40	**	*	*p* = 0.951 (NS)	***

By month 3, root numbers at depth (20–40 cm) were higher than within the upper 20 cm in all grasses, except for Bx509 (where significantly high root numbers, more than the other grasses studied, were found in similar numbers throughout the soil column) and AberStar (which maintained a higher root frequency in the upper 20 cm throughout the 4‐month study) (Figure 1). Root ontogeny of the three *Festulolium* Bx510, Bx511, and Bx514 was similar throughout, with root numbers recorded within the 20–40 cm region in excess of those found in the upper 20 cm by month 3. Previous to month 3, more roots were found within the upper 20 cm region of the soil column in all three *Festulolium* populations.

### 3.1 Comparisons of ontogeny found in Festulolium populations and their ryegrass controls (Tables 2, 3, 4)

**Table 2:** Comparison of root number between *Festulolium* introgression lines Bx509 and Bx510 (both 2n = 2x = 14) and root numbers found in *L. perenne* cv AberStar (2n = 2x = 14) throughout 0–20 and 25–40 cm soil profiles. Grasses with more roots are indicated (*p* < 0.05*; *p* < 0.01**; *p* < 0.001***; *p* < 0.0001****, NS = not significant)

Grass	Soil depth (cm)	Month 1	Month 2	Month 3	Month 4
Bx509	0–20	****	****	****	*p* = 0.058 (NS)
	20–40	*p* = 0.078 (NS)	****	****	****
Bx510	0–20	*p* = 0.075 (NS)	**	*p* = 0.938	*p* = 0.770 (NS)
	20–40	*p* = 0.608 (NS)	****	*****	*p* = 0.265 (NS)

**Table 3:** Comparison of root number between *Festulolium* introgression lines Bx511 and Bx514 (both 2n = 4x = 28) and root numbers found in *L. perenne* cv AberBite (2n = 4x = 28) throughout 0–20 cm and 25–40 cm soil profiles. Grasses with more roots are indicated (*p* < 0.05*; *p* < 0.01**; *p* < 0.001***; *p* < 0.0001***, NS = not significant)

Grass	Soil depth (cm)	Month 1	Month 2	Month 3	Month 4
Bx511	0–20	*p* = 1 (NS)	*p* = 0.367 (NS)	*p* = 0.681 (NS)	*
	20–40	*p* = 1 (NS)	*p* = 0.450 (NS)	*p* = 0.136 (NS)	***
Bx514	0.20	*p* = 0.880 (NS)	*p* = 0.133 (NS)	*p* = 0.493 (NS)	*p* = 0.789 (NS)
	20–40	*p* = 1 (NS)	*p* = 1 (NS)	*p* = 0.341 (NS)	**

**Table 4:** Mean leaf dry weights (DMY) of individual plant genotypes of six grass varieties or populations (based on three replicates; 20 genotypes/variety or population) from cuts made from root columns immediately preceding root imaging. *Festulolium* grasses with significantly higher yield (<0.05%) than their *L. perenne* controls indicated*

Grass	Cut 1 (gm)	Cut 2 (gm)	Cut 3 (gm)	Cut 4 (gm)
Lp cv AberStar (2×)	1.18	3.5	5.23	5.81
Bx509 (2×)	3.01*	7.02*	6.47*	6.65
Bx510 (2×)	2.20*	5.73*	5.62	4.48
Lp cv AberBite (4×)	1.80	3.84	5.33	5.27
Bx 511	1.74	4.75	6.36	5.94
Bx 514	1.92	4.67	6.36	5.10
l.s.d (*p* < 0.05)	0.37	0.94	1.17	1.44

#### 3.1.1 Bx509 v L. perenne cv AberStar (2x)

Differences over four consecutive months in root number at 0–20 and 20–40 cm depths between the drought‐tolerant introgression line Bx509 and its diploid ryegrass parent AberStar are presented in Table 2 and illustrated in Figure 1. At 0–20 cm, significantly more roots were present in Bx509 over the first 3 months but by month 4, root numbers between Bx509 and AberStar were not different significantly. At 20–40 cm, root numbers although initially not significantly different, in Bx509, had by month 2 increased and then remained consistently significantly higher over the subsequent 2 months.

The mean/genotype forage yield of Bx509 was significantly higher (*p* < 0.05) than *L. perenne* cv AberStar over the first 3 months but was not significantly different at month 4 (Table 4).

#### 3.1.2 Bx510 v L. perenne cv AberStar (2x)

Differences in root development between drought‐tolerant Bx510 and its ryegrass parent AberStar were not as evident as in Bx509. At 0–20 cm, more roots were present in Bx510 in month 2 but differences in root number were not significant(NS) in the other months assessed, and at 20–40 cm, root number in Bx510 was greater in number than AberStar in months 2 and 3 (Table 2; Figure 1).

The mean/genotype forage yield of Bx510 was significantly higher (*p* < 0.05) than *L. perenne* cv AberStar over the first 2 months but was not significantly different over the remaining 2 months (Table 4).

#### 3.1.3 Bx511 v L. perenne cv AberBite (4x)

Differences in root number in the allotetraploid hybrid Bx511 and autotetraploid cv AberBite were not significantly different, near the soil surface (0–20 cm) or at depth (20–40 cm) over the first 3 months, but root numbers in Bx511 were significantly higher than of AberBite throughout all depths in the soil column by month 4 (Table 3; Figure 1).

The mean/genotype forage yield of Bx511, although higher over months 2–4, was not different significantly to that of *L. perenne* cv AberBite over any of the four consecutive months of root scans (Table 4).

#### 3.1.4 Bx514 v L. perenne cv AberBite (4x)

Root numbers in allotetraploid Bx514 and cv AberBite were not significantly different in the upper 20 cm throughout the 4 months of growth. Likewise, they were not significantly different at depth (20–40 cm) over the first 3 months but by month 4 Bx514 had significantly increased root frequency compared to the ryegrass control (Table 3; Figure 1).

The mean/genotype forage yield of Bx514 was similar over all four monthly harvests to Bx511 and similarly was not significantly different to *L. perenne* cv AberBite (Table 4).

## 4 DISCUSSION

Assessment and development of new forage grass varieties have progressed largely without consideration given to the extent of their root development. While *Festulolium* hybrids are considered generally to have deeper root systems than *Lolium* varieties, which may confer them some selective advantage when exposed to water‐limiting conditions (Durand et al., 2007), comparisons undertaken have been limited to small experimental genotype numbers and have not incorporated comparisons in root development throughout the growing season. Prior to the current research, analysis of root ontogeny at IBERS had relied on the use of root columns with visual scores taken at the start and end of a growing season. Notwithstanding these limitations, these investigations led to some significant conclusions in terms of positive measures of impact of *Festulolium* root ontogeny on ecosystem services when compared to ryegrass growing under equivalent conditions (MacLeod et al., 2013). They were also achieved over a much greater time period (representing two consecutive growing seasons) than was used in the current research. However, the research presented here provides for the first time a statistically rigorous comparison between root growth achieved in two diploid *Festulolium* introgression lines and two amphiploid hybrid populations and *Lolium* controls, chosen for their close genetic background, over four consecutive months. The outcomes indicate the efficacy of the NPPC LemnaTec‐based image technologies for measures of grass root ontogeny and analysis of aspects of root architecture in perennial forage grasses that can be applied at a scale suitable for surveying genotype differences in a quantitative and objective manner.

Targets for grass breeding programs should include both aboveground and belowground traits in order to achieve optimal field performance. In the current work, a close association at the four harvests of aboveground forage yield with the root growth recorded in the six grass populations was seen. This was particularly evident in the comparisons made between the diploid *Festulolium* populations Bx509 and Bx510 and their control variety, *L. perenne* cv AberStar. Bx509 had significantly higher root number and forage yield than AberStar in months 1–3. Similarly, Bx510 had significantly higher root number and forage yields than AberStar in months 1–2. The forage yields of the tetraploid *Festulolium* populations Bx511 and Bx514 were not significantly different to that of their *L. perenne* control, cv AberBite. It might be inferred from the current research that root frequency in *Lolium* and *Festulolium* could be predicted based on measures of foliar yield. However, root architecture and ontogeny in these grasses, as shown by MacLeod et al. (2013), have important implications on soil structure, hydrology, biota, and ecosystem services. Moreover, variants to root architecture may bring alternative benefits associated with crop production. For example, shallow roots are beneficial where there is limited phosphorus (Clark et al., 2011; Ho, Rosas, Brown, and Lynch, 2005). Kell (2011) suggested that breeding crop plants with deeper and prolific root ecosystems could simultaneously improve the soil structure and its steady‐state carbon, water and nutrient retention, as well as assisting increased crop production.

Many studies have found that deeper rooting may contribute to drought tolerance in the field (Fang et al., 2013; Zhu, Ingram, Benfey, & Elich, 2011). An increase in root number, particularly at depth, is considered a target for grass breeding programs, particularly due to climate change and onsets of increased periods of drought (Marshall et al., 2015). Durand et al. (2007) demonstrated in *Festulolium* genotypes an enhanced capability to extract water at depth in comparison with *Lolium* due to their deeper root systems. Plant rooting depth and penetration of soil are far more complex than mere possession of the genetic potential for deep root production and will be affected greatly by soil depth, soil type, and texture, and by bulk density and compaction. In many cereal crops, root elongation is reduced in proportion to the mechanical impedance of soils with significant reductions found following 2 weeks of growth when subjected to compacted soils (Ehlers, Kopke, Hesse, & Bohm, 1983; Goodman & Ennos, 1999; Merotto & Mundstock, 1999). Studies in rice have identified quantitative trait loci (QTL) that are associated with differential soil penetration that shows a trade‐off between root thickness and length (Price, Steele, Moore, Barraclough, & Clark, 2000). Whalley et al. (unpublished) used wax layers to simulate compacted soils with the aim of identifying QTL for improved root strength within a *Festulolium* mapping population. (Harper et al., 2011) found a monosomic chromosome substitution line where a single *L. perenne* chromosome 3 was replaced by its *F. pratensis* homologue to be one potential source of genes for selection of grasses with improved root strength. Chromosome 3 in the *Lolium‐Festuca* genome complex is syntenic with rice chromosome 1 which is known to carry a QTL for root strength (Price et al., 2000).

Alm et al. (2011) using a *F. pratensis* mapping population identified a large QTL on linkage group 3 for survival against severe droughts that was not apparent in a *L. perenne* mapping population described by Turner et al. (2008). Chromosome 3 in fescue species has proven to be an excellent source of novel alleles for drought resistance when transferred to ryegrass. Humphreys et al. (2006) demonstrated how alternative QTL for drought resistance derived from *F. arundinacea* and from *F. arundinacea var glaucescens* had been transferred onto different locations of *L. multiflorum* chromosome 3. The incorporation of the fescue introgression had in both cases enhanced the water‐use efficiency of the ryegrass significantly (Humphreys et al., 2012). The identical fescue introgression has been subsequently transferred into *L. perenne* through marker‐assisted selection (Marshall et al., 2015) to give rise to the two populations Bx509 and Bx510 studied herein. Both of these populations demonstrate an improved water‐use efficiency compared to that of *L. perenne* cv AberStar (Marshall et al., 2015). Compared to AberStar and among the four *Festulolium* populations studied here, Bx509 is shown to have significantly greater root numbers near to the soil surface (0–20 cm), and especially at depth (20–40 cm). It would be logical that the deep root system found in Bx509 would be a significant contributor to its high water‐use efficiency when confronted with drought conditions. The fescue introgression in Bx509 was derived from *F. arundinacea*, a fescue species known for its deep rooting and drought resistance (Cougnon et al., 2017), and was translocated onto an intercalary location on the long arm of chromosome 3 of *Lolium*.

The drought‐tolerant introgression line Bx510, although with lower root number than Bx509, had a deeper root system than AberStar by month 3 which would aide better water acquirement under water‐limiting conditions. In Bx510, the translocated fescue introgression derived from *F. arundinacea var glaucescens* and located at a terminal position on the satellite region of chromosome 3 of *Lolium* (Humphreys et al., 2005). Its role in improving water‐use efficiency may well differ from those in Bx509.

Irrespective of the potential for root growth at depth, deeper roots are generally confined to soil pores (Lynch & Wojciechowski, 2015). Gao et al. (2016) provided an explanation related to the increased soil penetrometer resistance that occurs with depth, even in soils that have not been damaged by compaction, although the effects are exacerbated by compaction. They suggest that root penetration into deep soil layers is likely to depend on the roots’ abilities to find existing pore networks, and consequently, soil structure and its management are major factors affecting capability for deep rooting. The successful colonization of soil at depth by a grass will require both a genetic capacity and incorporation of a suitable soil type and management regime. In the study undertaken here, noncompacted porous compost in soil columns was used throughout, which should largely eliminate any potential soil constraints on root growth, indicating the differences found between root growth in *Lolium* and *Festulolium* populations had a genetic basis.

Cougnon et al. (2017) undertook a detailed comparison of root biomass at soil depths of 0‐90 cm of *F. arundinacea, F. pratensis, L. perenne,* and two *Festulolium* varieties Achilles (*L. multiflorum *×* F. pratensis*) and Lueur (*L. multiflorum *×* F. arundinacea var glaucescens*) when grown in a sandy loam soil under two contrasting N fertilizer applications. The root biomass scores taken in the autumn of the third year of a field plot trial showed more roots at depth in *F. arundinacea* than in the other grasses but differences with Lueur were not significant (*p* > 0.05). Lueur shares a common *F. arundinacea var glaucescens* genome with Bx514 used in the current study but has a different *Lolium* species parent. The alternative approaches to root measures in forage grasses used by Cougnon et al. (2017) and used herein demonstrate the contrasting strengths and weaknesses of both approaches. The current root image analysis has the advantage of providing more temporal precision of root ontogeny found in *Festulolium* and *Lolium* populations and discriminates clear genetic effects. However, in contrast to the methods of Cougnon et al. (2017), the root imaging methods used here took little or no account of soil effects and of “real field” conditions with grass roots confined to 12‐cm‐wide and 50‐cm‐deep soil columns. Prior to future grass variety advancement, it will be necessary to confirm that advantageous root designs identified by dynamic imaging are replicated when grass populations are sown as crops and grown under field conditions.

The *Festulolium* amphiploid hybrids Bx514 and especially Bx511, which contains genomes of *F. mairei*, had greater root numbers particularly at depth than their tetraploid *Lolium* control, AberBite by month 4. Their agronomic potential has been described previously, including the opportunities they provide for increasing efficiency in ruminant nutrition (Humphreys et al., 2014). Moreover, their root–soil interactions are currently being explored for their potential use in flood mitigation within the BBSRC‐LINK SureRoot project. The project was designed to validate earlier findings whereby a *Festulolium* variety Prior (an amphiploid hybrid between *L. perenne* × *F. pratensis*) was found to have altered soil structure and enhanced soil porosity to an extent where rainfall runoff was reduced by 51% compared to grass plots containing *L. perenne* (MacLeod et al., 2013). The suggested explanation provided by MacLeod et al. (2013) was that the deep rooting of *Festulolium* cv Prior and its later senescence, especially at depth, had enhanced soil porosity and soil water retention.

In a recent modeling study of ecosystem services associated with the SureRoot project using data taken from fields of *Festulolium* cv Prior and of *L. perenne* cv AberMagic, Li et al. (2017) cv Prior was predicted to have potential to fix more and lose less C through soil respiration than *L. perenne cv* AberMagic. The authors suggested the ability to fix C might be due to the larger and proportionately deeper root biomass. Simulations suggested that soil C storage with reseeded cv Prior gradually increased by 0.525 t C/ha in 2 years following reseeding. Furthermore, in comparison with permanent pasture, both cvs AberMagic and Prior reduced the N losses through runoff and contributed to reducing water loss, especially *Festulolium* cv Prior in relation to the latter.

The accumulated evidence from research such as that of MacLeod et al. (2013) and Li et al. (2017) would suggest that root turnover in certain *Festulolium* hybrids may have a significant impact on increasing soil porosity, be causal in soil water retention, and may mitigate incidents of flooding as well as impacting on carbon sequestration. However, the impacts of plant roots over soil structure have alternative explanations. Bardgett et al. (2014) considered the root trait that has the greatest impact on soil structure is root exudation, which increases soil aggregate stability. This is because root exudates contain polysaccharides and proteins which bind mineral particles together and more importantly promote the abundance of microorganisms in the rhizosphere which restructure and stabilize soils at the microscale (Brown, George, Neugebauer, & White, 2017).

It is clear for future grass designs to become more holistic and to capture their full potential for combined excellent agronomic performance and additional valuable ecosystem services that inclusions of dynamic imaging for precision measures of root growth such as those reported here will provide great benefit.

## CONFLICT OF INTEREST

None declared.
